# Molecular phylogenetic evidence supports a new family of octocorals and a new genus of Alcyoniidae (Octocorallia, Alcyonacea)

**DOI:** 10.3897/zookeys.346.6270

**Published:** 2013-11-01

**Authors:** Catherine S. McFadden, Leen P. van Ofwegen

**Affiliations:** 1 Department of Biology, Harvey Mudd College, 1250 N. Dartmouth Ave., Claremont, CA 91711, USA; 2 Department of Marine Zoology, Naturalis Biodiversity Center, P.O. Box 9517, 2300 RA Leiden, The Netherlands

**Keywords:** *Alcyonium*, *Eleutherobia*, Parasphaerascleridae, *Parasphaerasclera*, *Sphaerasclera*, Indo-Pacific, taxonomy

## Abstract

Molecular phylogenetic evidence indicates that the octocoral family Alcyoniidae is highly polyphyletic, with genera distributed across Octocorallia in more than 10 separate clades. Most alcyoniid taxa belong to the large and poorly resolved Holaxonia–Alcyoniina clade of octocorals, but members of at least four genera of Alcyoniidae fall outside of that group. As a first step towards revision of the family, we describe a new genus, *Parasphaerasclera* **gen. n.**, and family, Parasphaerascleridae **fam. n.**, of Alcyonacea to accommodate species of *Eleutherobia* Pütter, 1900 and *Alcyonium* Linnaeus, 1758 that have digitiform to digitate or lobate growth forms, completely lack sclerites in the polyps, and have radiates or spheroidal sclerites in the colony surface and interior. Parasphaerascleridae **fam. n.** constitutes a well-supported clade that is phylogenetically distinct from all other octocoral taxa. We also describe a new genus of Alcyoniidae, *Sphaerasclera* **gen. n.**, for a species of *Eleutherobia* with a unique capitate growth form. *Sphaerasclera* **gen. n.** is a member of the *Anthomastus–Corallium* clade of octocorals, but is morphologically and genetically distinct from *Anthomastus* Verrill, 1878 and *Paraminabea* Williams & Alderslade, 1999, two similar but dimorphic genera of Alcyoniidae that are its sister taxa. In addition, we have re-assigned two species of *Eleutherobia* that have clavate to capitate growth forms, polyp sclerites arranged to form a collaret and points, and spindles in the colony interior to *Alcyonium*, a move that is supported by both morphological and molecular phylogenetic evidence.

## Introduction

The anthozoan sub-class Octocorallia comprises a clade of approximately 350 genera and 3400 species of soft corals, gorgonians and sea pens that are found throughout marine environments worldwide (Daly et al. 2007; Williams and Cairns 2013). The current morphology-based taxonomic classification of Octocorallia recognizes three orders, with the majority of families and species belonging to Alcyonacea Lamouroux, 1816 (soft corals, gorgonians and stoloniferans) (Daly et al. 2007). Attempts to further subdivide this very large taxon into smaller orders or sub-ordinal groups have been fraught with difficulty (McFadden et al. 2010). Molecular phylogenetic reconstructions of Octocorallia have confirmed that most of the morphologically defined sub-ordinal groups that have traditionally been recognized (Bayer 1981, Fabricius and Alderslade 2001)—as well as a majority of families—represent polyphyletic assemblages (Berntson et al. 2001; McFadden et al. 2006, 2010; McFadden and Ofwegen 2012). Analyses based on mitochondrial genes (McFadden et al. 2006), nuclear ribosomal genes (Berntson et al. 2001), and both (Brockman and McFadden 2012, McFadden and Ofwegen 2012) instead support the division of Octocorallia into two major clades, Holaxonia–Alcyoniina and Calcaxonia–Pennatulacea, plus a third, smaller clade, *Anthomastus*–*Corallium* (McFadden et al. 2006, 2010). The phylogenetic relationships among the family-level clades that comprise the morphologically heterogeneous mix of soft corals (Alcyoniina), gorgonians (Holaxonia, Scleraxonia) and stoloniferous forms (Stolonifera) belonging to Holaxonia–Alcyoniina remain unresolved (McFadden et al. 2006, 2010), hindering efforts to revise their taxonomy.

Among the many families of octocorals that appear from molecular phylogenetic analyses to be polyphyletic, the soft coral family Alcyoniidae stands out as one of the most phylogenetically heterogeneous (McFadden et al. 2006, 2010). Alcyoniidae is also a morphologically eclectic taxon, as it has frequently been the repository for genera that lack the specific diagnostic characters of other, more narrowly circumscribed families (Daly et al. 2007). Genera of Alcyoniidae are distributed across Octocorallia in at least 10 distinct clades, with the majority belonging to the poorly resolved Holaxonia–Alcyoniina clade (McFadden et al. 2006). Although the family clearly requires taxonomic revision, increased phylogenetic resolution along the backbone of Holaxonia–Alcyoniina will be necessary in order to determine which clades of Alcyoniidae should be reassigned to different families, and what the diagnostic morphological characters of those families might be.

Several genera of Alcyoniidae fall entirely outside of Holaxonia–Alcyoniina, and belong instead to two small clades located near the base of Octocorallia (McFadden et al. 2006). These include *Anthomastus* Verrill, 1878 and *Paraminabea* Williams & Alderslade, 1999, both of which belong to the *Anthomastus*–*Corallium* clade (McFadden et al. 2006, Brockman and McFadden 2012). Sufficient molecular phylogenetic evidence now exists to suggest that several species in the alcyoniid genera *Eleutherobia* Pütter, 1900 and *Alcyonium* Linnaeus, 1758 comprise a second clade that also lies outside of Holaxonia–Alcyoniina and is well separated from all other genera of Alcyoniidae. Here we present corroborating morphological evidence to support the description of a new family and genus of Alcyonacea to accommodate this unique clade. In addition, we designate a new genus and combination for a species of *Eleutherobia* that belongs to the *Anthomastus*–*Corallium* clade, and reassign two other species of *Eleutherobia* to *Alcyonium*.

## Methods

### Collection

Specimens of *Eleutherobia* and *Alcyonium* suitable for molecular analyses were collected in South Africa in 2008 and Palau in 2005 and 2010 (Table 1). Following collection using SCUBA, colonies were preserved in 70% EtOH; tissue sub-samples to be used for molecular analyses were preserved in >90% EtOH. Specimens of *Eleutherobia flammicerebra* Williams, 2003 were collected by dredge from New Caledonia during the 2008 Terrasses cruise (R/V Alis), conducted as part of the MNHN/IRD Tropical Deep-Sea Benthos cruises, 2003–2012 (Bouchet et al. 2008). Vouchers have been deposited at the Naturalis Biodiversity Center (formerly Rijksmuseum van Natuurlijke Historie, Leiden (RMNH)), and the Muséum national d’Histoire naturelle, Paris (MNHN). Additional material was obtained from the Museum and Art Gallery of the Northern Territory, Darwin, Australia (NTM), the Florida Natural History Museum, Gainesville (UF), the Zoological Museum, University of Copenhagen (ZMUC), and the Zoologische Staatsammlung München, Germany (ZSM) (Table 1).

**Table 1.** Specimens of *Alcyonium*, *Parasphaerasclera* gen. n. and *Sphaerasclera* gen. n. included in molecular phylogenetic and morphological analyses. For GenBank accession numbers see Appendix. For abbreviations, see Methods section.

Genus & Species	Authority	Museum & Cat. No.	Collection Location	Year
***Alcyonium***	Linnaeus, 1758			
*Alcyonium bocagei*	(Saville Kent, 1870)	RMNH Coel. 39672	Portugal, Sagres	1994
*Alcyonium coralloides*	(Pallas, 1766)	RMNH Coel. 39678	France, Marseilles	1994
*Alcyonium digitatum*	Linnaeus, 1758	RMNH Coel. 39671	Isle of Man	1991
*Alcyonium glomeratum*	(Hassall, 1841)	RMNH Coel. 39668	France, Iles des Glenans	1994
*Alcyonium haddoni*	Wright & Studer, 1889	ZSM 20061191	Chile, Canal Pitt Chico	2006
*Alcyonium hibernicum*	Renouf, 1931	RMNH Coel. 39661	Isle of Man	1991
*Alcyonium palmatum*	Pallas, 1766	RMNH Coel. 39685	NE Spain	1996
*Alcyonium varum*	McFadden & Ofwegen, nom. n.	ZSM 20061195	Chile, Paso del Abismo	2006
*Alcyonium sidereum*	Verrill, 1922		USA, Massachusetts	1989
*Alcyonium variabile*	(Thomson, 1921)	RMNH Coel. 40800	South Africa, Algoa Bay	1998
*Alcyonium variabile*	(Thomson, 1921)	RMNH Coel. 41530	South Africa, Algoa Bay	2008
*Alcyonium variabile*	(Thomson, 1921)	RMNH Coel. 41531	South Africa, Algoa Bay	2008
***Parasphaerasclera* gen. n.**				
*Parasphaerasclera aurea*	(Benayahu & Schleyer, 1995)	RMNH Coel. 40205	South Africa, Park Rynie	2008
*Parasphaerasclera aurea*	(Benayahu & Schleyer, 1995)	RMNH Coel. 40799	South Africa, Park Rynie	2008
*Parasphaerasclera aurea*	(Benayahu & Schleyer, 1995)	RMNH Coel. 41535	South Africa, Aliwal Shoal	2008
*Parasphaerasclera* aff. *grayi*	(Thomson & Dean, 1931)	NTM C14092	Palau, Babeldaob	2005
*Parasphaerasclera* aff. *grayi*	(Thomson & Dean, 1931)	RMNH Coel. 40920	Palau, Pelelieu	2010
*Parasphaerasclera rotifera*	(Thomson, 1910)	UF3890	South Africa, East London	1999
*Parasphaerasclera valdiviae*	(Kükenthal, 1906)	RMNH Coel. 40206	South Africa, Algoa Bay	2008
*Parasphaerasclera valdiviae*	(Kükenthal, 1906)	RMNH Coel. 41532	South Africa, Algoa Bay	2008
*Parasphaerasclera valdiviae*	(Kükenthal, 1906)	RMNH Coel. 41534	South Africa, Algoa Bay	2008
***Sphaerasclera* gen. n.**				
*Sphaerasclera flammicerebra*	(Williams, 2003)	MNHN-IK-2012-12004	New Caledonia	2008
*Sphaerasclera flammicerebra*	(Williams, 2003)	ZMUC-ANT-000256	Mauritius	1929

### Morphological analysis

Sclerites were obtained by dissolving tissues from the upper and lower regions of a colony in 10% sodium hypochlorite (household bleach). Sclerites were rinsed well with deionized water, dried, and mounted on stubs for SEM. They were imaged using a JEOL JSM-6480LV scanning electron microscope operated at 10 kV.

### Molecular phylogenetic analyses

Extraction of DNA from ethanol-preserved tissue samples, PCR amplification, and sequencing of the *mtMutS* (*msh1*) and *COI* genes followed the protocols published in McFadden et al. (2011). In addition, we sequenced an approximately 810 nt fragment of the 28S nuclear ribosomal gene using primers 28S-Far and either 28S-Rar or 28S-Rab (McFadden and Ofwegen 2013). Sequence data were aligned to a previously compiled reference dataset of 130 octocorals and anthozoan outgroup taxa (McFadden and Ofwegen 2012; Appendix) using the L-INS-i method in MAFFT (Katoh et al. 2005). Modeltest 3.0 (Posada and Crandall 1998) was used to select appropriate models of evolution for maximum likelihood analyses that were run for 100 bootstrap replicates using GARLI 2.0 (Zwickl 2006). The 28S rDNA and mitochondrial gene (*mtMutS* + *COI*) datasets were analyzed separately, and in a combined analysis with different models of evolution applied to separate data partitions (mt genes: TVM+I+G; 28S: GTR+I+G). Bayesian analyses were run using MrBayes v. 3.2.1 (Ronquist et al. 2012) with the same data partitions; because MrBayes does not support the TVM model, however, a GTR+I+G model was applied separately to each partition. Analyses were run for 6,000,000 generations (until runs had converged and standard deviation of split partitions < 0.01) and sampled every 500 generations, with a burn-in of 25% and default Metropolis coupling parameters.

## Results

### Molecular phylogenetic analyses

**Figure 1.**
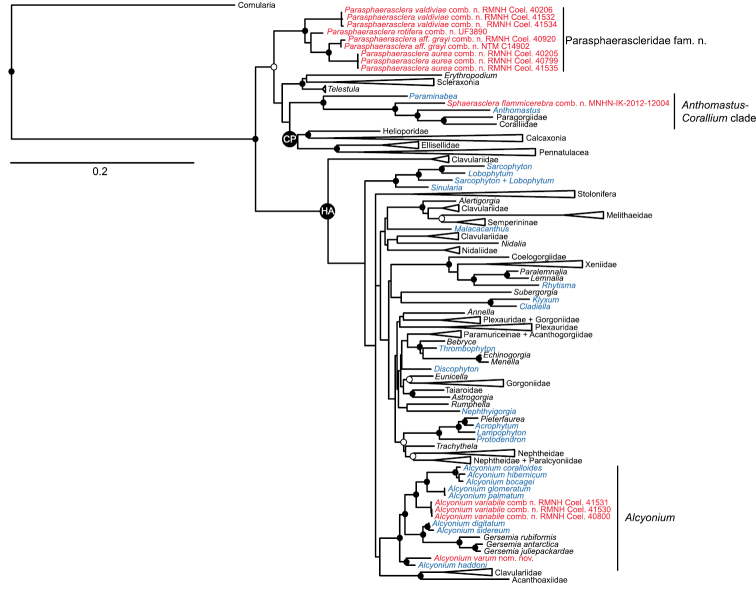
Maximum likelihood tree of Octocorallia based on combined, partitioned analysis of *mtMutS*, *COI* and 28S rDNA sequences. Taxa belonging to family Alcyoniidae are shown in blue; new combinations proposed herein are shown in red. Solid circles at nodes indicate strong support from both maximum likelihood (bootstrap values >70%) and Bayesian inference (posterior probability > 0.95); open circles indicate moderate support (bootstrap values >50%, Bayesian pp > 0.95). Strongly supported clades that include no alcyoniid taxa have been collapsed to triangles to facilitate readability. HA: Holaxonia–Alcyoniina clade; CP: Calcaxonia–Pennatulacea clade. Hexacorallian taxa used as outgroups are not shown. For a list of all reference taxa and sequences included in the analysis see Appendix.

The separate maximum likelihood analyses of the 28S rDNA and mitochondrial gene alignments generated phylogenies that were congruent with one another (with the sole exception of some internal relationships within Pennatulacea), but were poorly resolved overall. The partitioned analyses of the combined mt + 28S dataset had much higher support values for many of the deeper nodes within the tree, and it is these analyses that we present (Fig. 1) and discuss here. As has been demonstrated previously based on analyses of similar datasets (Brockman and McFadden 2012, McFadden and Ofwegen 2012), the combined gene analyses supported the division of the majority of octocorals among two major clades, Holaxonia–Alcyoniina and Calcaxonia–Pennatulacea (Fig. 1). In addition, there was strong support from both maximum likelihood and Bayesian methods for three small clades that fell outside of Holaxonia–Alcyoniina but whose relationships to Calcaxonia–Pennatulacea and to one another remain unresolved. These include the previously recognized *Anthomastus*–*Corallium* clade (McFadden et al. 2006, Brockman and McFadden 2012), a clade comprising a heterogeneous mix of scleraxonians plus the stoloniferan genus *Telestula* Madsen, 1944, and a clade consisting of several species of the alcyoniid genera *Eleutherobia* and *Alcyonium* (Fig. 1). The stoloniferan genus *Cornularia* Lamarck, 1816 was recovered as the sister taxon to all other octocorals.

Members of family Alcyoniidae were distributed throughout Holaxonia–Alcyoniina in nine distinct clades (Fig. 1). Some of these clades included only alcyoniid genera, while others supported close relationships among alcyoniids and genera classified in different families. There was insufficient resolution of the deeper nodes within Holaxonia–Alcyoniina to infer the phylogenetic relationships of any of these nine clades to one another. In addition, four genera of Alcyoniidae fell entirely outside of Holaxonia–Alcyoniina (Fig. 1). These included *Paraminabea* and *Anthomastus*, both of which belonged to the *Anthomastus*–*Corallium* clade, as well as several species of *Eleutherobia* and *Alcyonium* that formed a distinct, well-supported clade whose relationship to the Calcaxonia–Pennatulacea, *Anthomastus*–*Corallium* and Scleraxonia–*Telestula* clades was poorly resolved. This clade included *Eleutherobia aurea* Benayahu & Schleyer, 1995, *Eleutherobia rotifera* (Thomson, 1910), *Eleutherobia* aff. *grayi* (Thomson & Dean, 1931), and *Alcyonium valdiviae* Kükenthal, 1906. Two of the species of *Eleutherobia* we sequenced were not included in this clade: *Eleutherobia variabile* (Thomson, 1921) fell into a clade with northern hemisphere members of the genus *Alcyonium*, while *Eleutherobia flammicerebra* Williams, 2003 belonged to the *Anthomastus*–*Corallium* clade, phylogenetically distinct from both *Paraminabea* and *Anthomastus* (Fig. 1).

## Taxonomic section

### Alcyonacea Lamouroux, 1816

#### Alcyoniidae Lamouroux, 1812

##### *Alcyonium* Linnaeus, 1758

*Alcyonium* has long served as a repository for species that lack characters to support their placement in other more narrowly circumscribed genera. Over time the diagnosis of the genus has been broadened to include almost every possible colony growth form observed within Alcyoniidae (Williams 1988) as well as a diversity of different sclerite types and arrangements. In recent years the heterogeneity of this genus has been acknowledged (Alderslade 2000; Williams 2000a), and a number of new genera have been erected to accommodate species whose characters clearly differ from those of the type species, the northern hemisphere *Alcyonium digitatum* Linnaeus, 1758. Molecular phylogenetic analyses have supported the taxonomic distinction of new genera such as *Klyxum* Alderslade, 2000, *Rhytisma* Alderslade, 2000, *Lampophyton* Williams, 2000, and *Discophyton* McFadden & Hochberg, 2003 (Fig. 1), all of them established to accommodate species formerly placed in *Alcyonium*.

Phylogenetic evidence suggests that genus *Alcyonium* should be further restricted to species in which the polyp sclerites are arranged as a collaret and points and the sclerites of the coenenchyme are in two distinct layers, a surface layer consisting of predominantly radiates or clubs, and an inner layer of spindles or rods (Alderslade 2000). The colony growth form may be lobate, digitate, capitate or encrusting, and the sclerites are usually colored. Adoption of this restricted, phylogenetically supported diagnosis will necessitate not only the removal of additional species from *Alcyonium*, but also the inclusion of species currently placed in several other genera. For example, despite their placement in three different families, the genera *Bellonella* Gray, 1862 (Alcyoniidae), *Eleutherobia* Pütter, 1900 (Alcyoniidae), *Anthothela* Verrill, 1879 (Anthothelidae) and *Gersemia* Marenzeller, 1878 (Nephtheidae) all include species with sclerite characters that suggest a close affinity with *Alcyonium*. Molecular phylogenetic evidence supports a paraphyletic relationship between *Alcyonium*, *Gersemia*, and *Anthothela* (McFadden et al. 2006), as well as the inclusion of a species of *Eleutherobia* in *Alcyonium* *sensu stricto* (Fig. 1).

Williams (2003) reassigned *Alcyonium variabile* (Thomson, 1921) to *Eleutherobia* subsequent to his modification of that genus to accommodate the capitate *Eleutherobia flammicerebra* Williams, 2003. The capitate growth form of *Eleutherobia variabile* differs from the lobate to digitate forms characteristic of most northern hemisphere species of *Alcyonium* (McFadden et al. 2001). Similar to other species of *Alcyonium* *sensu stricto*, however, *Eleutherobia variabile* has polyp sclerites arranged to form a distinct collaret and points, radiates and club-like forms in the surface layer of the polyparium and stalk, and long spindles in the interior of the polyparium (Williams 1986, 1992). Molecular phylogenetic analyses strongly support the inclusion of *Eleutherobia variabile* in *Alcyonium* *sensu stricto* (Fig. 1), therefore we transfer this species back to that genus and re-instate the combination *Alcyonium variabile* (Thomson, 1921) comb. n.

*Bellonella studeri* Thomson, 1910, a species with a clavate to capitate growth form similar to that of *Alcyonium variabile* comb. n., was reassigned to *Eleutherobia* by Verseveldt and Bayer (1988). The sclerites of *Eleutherobia studeri* are very similar to those of *Alcyonium variabile* comb. n. Both species have polyps with spindles arranged to form a collaret and points, capstan-like radiates in the surface of the polyparium and stalk, and sclerites in the colony interior that are predominantly slender spindles (Williams 1992). Material of *Eleutherobia studeri* was not available for molecular phylogenetic analysis, but based on its morphological similarity to *Alcyonium variabile* comb. n. we suggest that this species also belongs in *Alcyonium*, and propose the new combination *Alcyonium studeri* (Thomson, 1910) comb. n. Verseveldt and Bayer (1988) suggested that Thomson’s (1910) *Metalcyonium clavatum* (non Pfeffer, 1889) might be a synonym of *Alcyonium studeri* comb. n. Whether or not that is the case, *Metalcyonium clavatum* also appears from Thomson’s (1910) description to belong to *Alcyonium*.

Our molecular phylogenetic analyses included a specimen of *Alcyonium roseum* Ofwegen, Häussermann & Försterra, 2007, a species recently described from Chile. We note that that name is pre-occupied by *Alcyonium roseum* (Tixier-Durivault, 1954), and hereby designate the Chilean species *Alcyoniumvarum* nom. nov. Etymology: from the Latin *varus*, crooked or bow-legged, denoting the shape of the sclerites in the polyps (Ofwegen et al. 2007).

##### Sphaerasclera gen. n.

http://zoobank.org/3DF0B00F-CB14-4B4C-A28A-0AF019EA35E4

http://species-id.net/wiki/Sphaerasclera

Figs 2 –3

###### Type species.

*Eleutherobia flammicerebra* Williams, 2003, by original designation.

###### Diagnosis.

Soft corals with a capitate growth form, with a distinct, spherical polyparium raised on a sterile stalk. Polyps monomorphic. Anthocodial sclerites absent. Sclerites of colony surface and interior are large, tuberculate spheroids and smaller radiates. Sclerites permanently colored. Azooxanthellate.

###### Etymology.

From the Latin/Greek *sphaera*- meaning a sphere or ball and Greek *sclero*-, hard, denoting the large, spheroidal sclerites that characterize this genus. Gender: fem.

###### Remarks.

**Figure 2.**
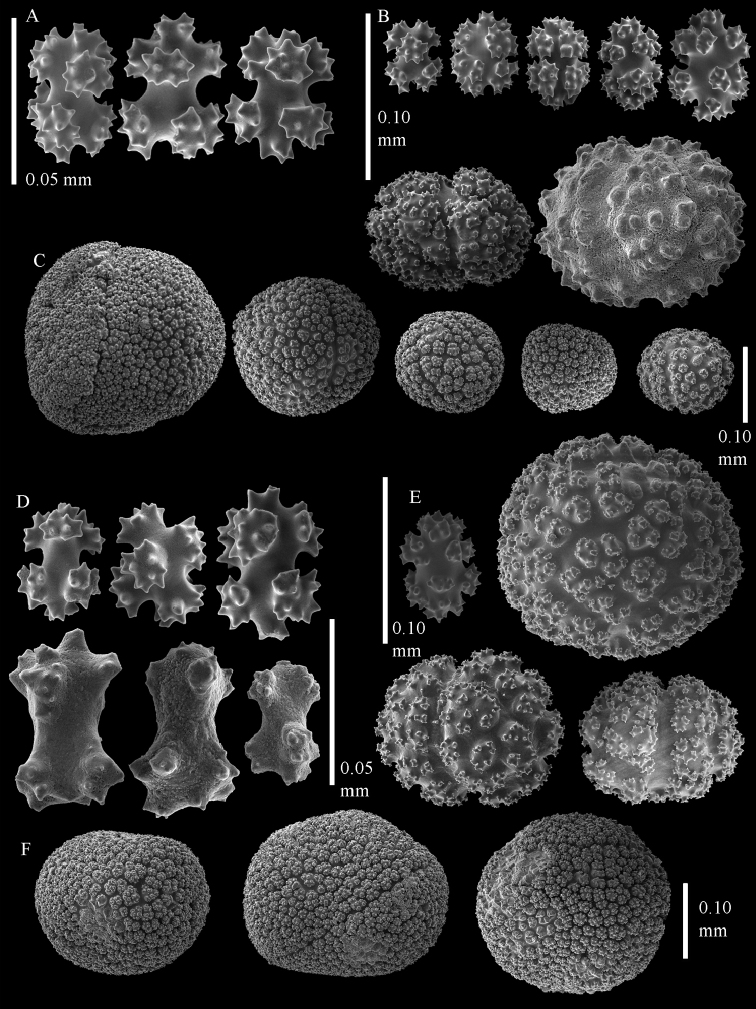
Sclerites of *Sphaerasclera flammicerebra* comb. n. MNHN-IK-2012-12004. **A–C** Surface layer of polyparium **A** Small radiates (0.05 mm scale bar) **B** Larger radiates and two small spheroids **C** Large tuberculate spheroids **D–F** Interior of polyparium **D** Small radiates (0.05 mm scale bar) **E** Larger radiate and three small spheroids **F** Large tuberculate spheroids.

**Figure 3.**
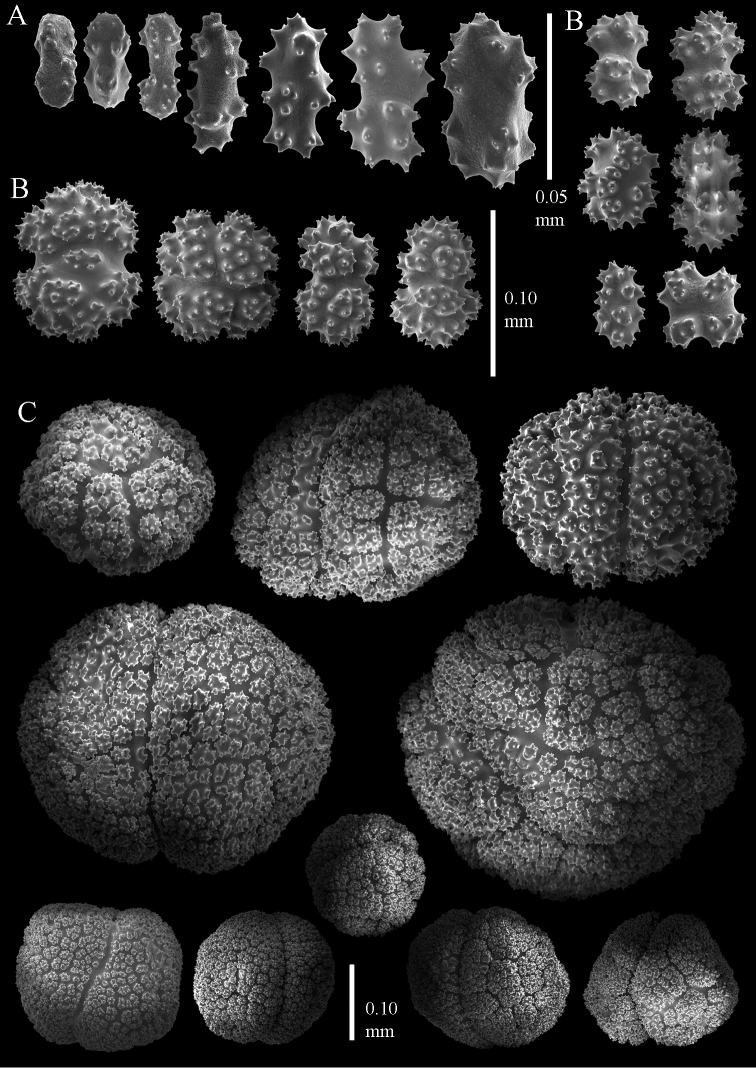
Sclerites of *Sphaerasclera flammicerebra* comb. n. ZMUC-ANT-000256. Sclerites of polyparium. **A**. Small radiates (left of 0.05 mm scale bar) **B** Larger radiates **C** Large tuberculate spheroids of colony surface and interior.

Williams’s (2003) assignment of *Eleutherobia flammicerebra* to *Eleutherobia* necessitated emending the diagnosis of that genus to include capitate growth forms. *Eleutherobia flammicerebra* does share other character states—such as monomorphic polyps that lack sclerites and tuberculate spheroids and radiates in the surface of the polyparium and stalk (Figs 2–3)—with some species of *Eleutherobia*. Molecular phylogenetic analyses suggest that *Eleutherobia flammicerebra* is, however, not closely related to morphologically similar members of *Eleutherobia* such as *Eleutherobia rotifera* (Thomson, 1910) but instead falls into the *Anthomastus*-*Corallium* clade of octocorals (Fig. 1). Based on its distinctive colony growth form and unique phylogenetic position, we hereby designate a new genus, *Sphaerasclera*, and new combination, *Sphaerasclera flammicerebra* (Williams, 2003) for this species.

Unlike all other members of the *Anthomastus*-*Corallium* clade, *Sphaerasclera* gen. n. appears to have monomorphic rather than dimorphic polyps. As discussed by Williams (2000b), however, siphonozooids may be difficult to detect in contracted, preserved material. The large, densely packed, spherical sclerites in the coenenchyme of *Sphaerasclera flammicerebra* comb. n. obscure the finer details of the colony surface, and also make this species very difficult to section for histological study of the polyps. For now we concur with Williams (2003) that the species is monomorphic, but entertain the possibility that future observation of living specimens might reveal the presence of siphonozooids.

Although *Sphaerasclera* gen. n. differs by having monomorphic polyps, it does share other morphological characters with *Paraminabea* and *Anthomastus*, the two genera with which it is most closely allied phylogenetically (Fig. 1). *Anthomastus* likewise includes species with capitate growth forms, but in that genus the autozooids have sclerites, and the sclerites in the surface and interior of the colony include rods and needles in addition to radiates (Bayer 1993). Like *Sphaerasclera* gen. n., *Paraminabea* lacks sclerites in the polyps and its coenenchymal sclerites are predominantly radiates and spheroids (Williams and Alderslade 1999). The colony growth form of *Paraminabea*, however, is digitiform, hemispherical or lobate rather than capitate. Moreover, *Paraminabea* has a unique molecular synapomorphy, a mitochondrial gene rearrangement that distinguishes it from all other genera of octocorals (Brockman and McFadden 2012). Screening of mitochondrial gene junctions suggests that *Sphaerasclera flammicerebra* comb. n. and *Anthomastus* both retain the ancestral octocoral mt gene order, and do not share the derived state found in *Paraminabea* (Brockman and McFadden 2012).

*Paraminabea* and *Anthomastus* are both classified in family Alcyoniidae, and for convenience we have also assigned *Sphaerasclera* gen. n. to that family. All other genera of Alcyoniidae, however, belong to the Holaxonia–Alcyoniina clade of Octocorallia, far removed phylogenetically from the *Anthomastus*-*Corallium* clade (Fig. 1). In addition to *Paraminabea*, *Anthomastus* and *Sphaerasclera* gen. n., the *Anthomastus*-*Corallium* clade also includes all members of Coralliidae Lamouroux, 1812 and Paragorgiidae Kükenthal, 1916, two families of gorgonians that have historically been assigned to the sub-ordinal group Scleraxonia. Although it is clear from the phylogenetic evidence that the soft coral taxa that fall within this clade should be removed from Alcyoniidae, we defer their reassignment to another family pending an in-depth analysis of the morphological character states shared among the seemingly heterogeneous genera and families that are united within *Anthomastus*-*Corallium*.

Our records extend the known geographic distribution of *Sphaerasclera flammicerebra* comb. n. from Palau (Williams 2003) to New Caledonia and Mauritius. The colony growth forms and sclerites of specimens from these widespread localities closely match that of the type material from Palau (Figs 2–3).

#### Parasphaerascleridae fam. n.

http://zoobank.org/82E6711B-C21A-4EE4-828E-F4EBBC4CA7BD

http://species-id.net/wiki/Parasphaerascleridae

##### Type genus.

*Parasphaerasclera* McFadden & Ofwegen, gen. n.

##### Included genera.

*Parasphaerasclera* gen. n.

##### Diagnosis.

Soft corals with a digitiform, digitate or lobate growth form, usually with a sterile stalk although this may be indistinct. Polyps monomorphic. Permanent calyces absent, although retracted polyps may remain visible as small mounds on the polyparium surface. Anthocodial sclerites absent. Sclerites of colony surface and interior predominantly radiates and tuberculate spheroids, occasionally rods and crosses. Sclerites permanently colored. Azooxanthellate.

##### Remarks.

**Figure 4.**
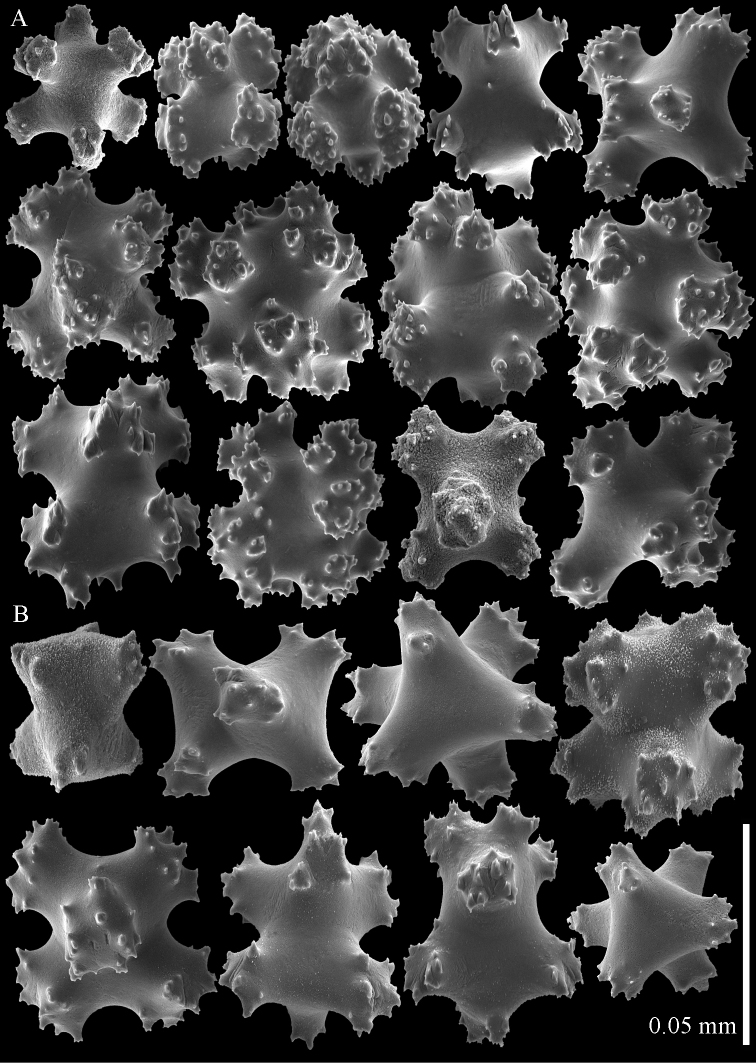
Sclerites of *Parasphaerasclera aurea* comb. n., RMNH Coel. 40779. **A** Surface of polyparium **B** Interior of polyparium.

**Figure 5.**
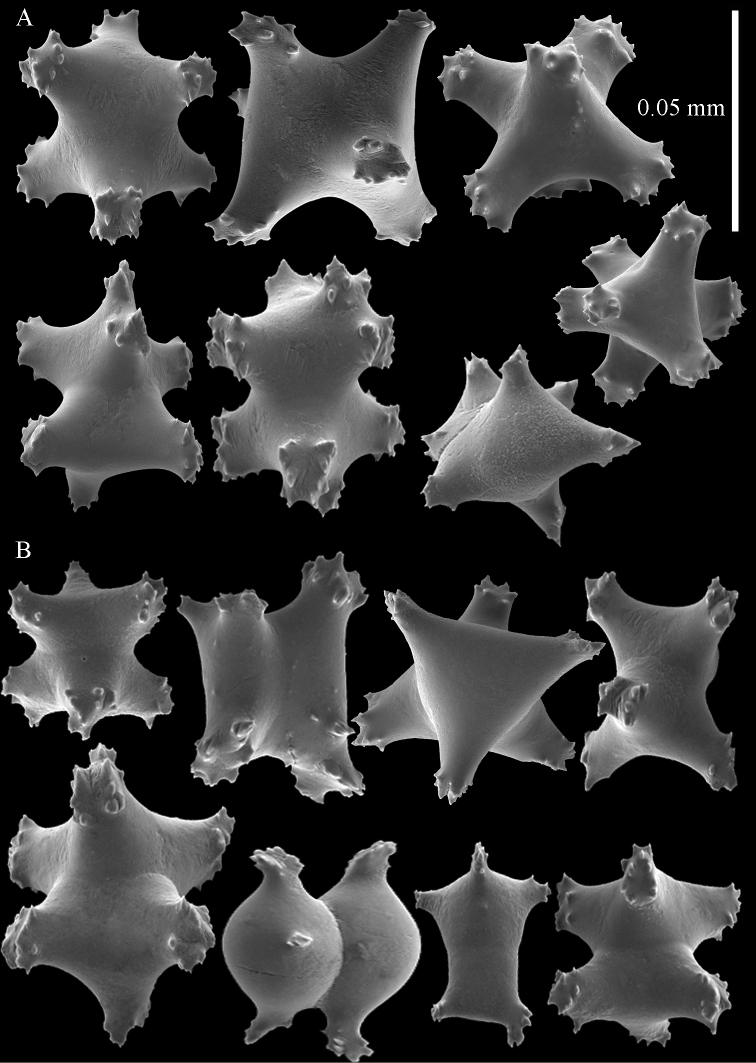
Sclerites of *Parasphaerasclera aurea* comb. n., RMNH Coel. 40779. **A** Surface of stalk **B** Interior of stalk.

**Figure 6.**
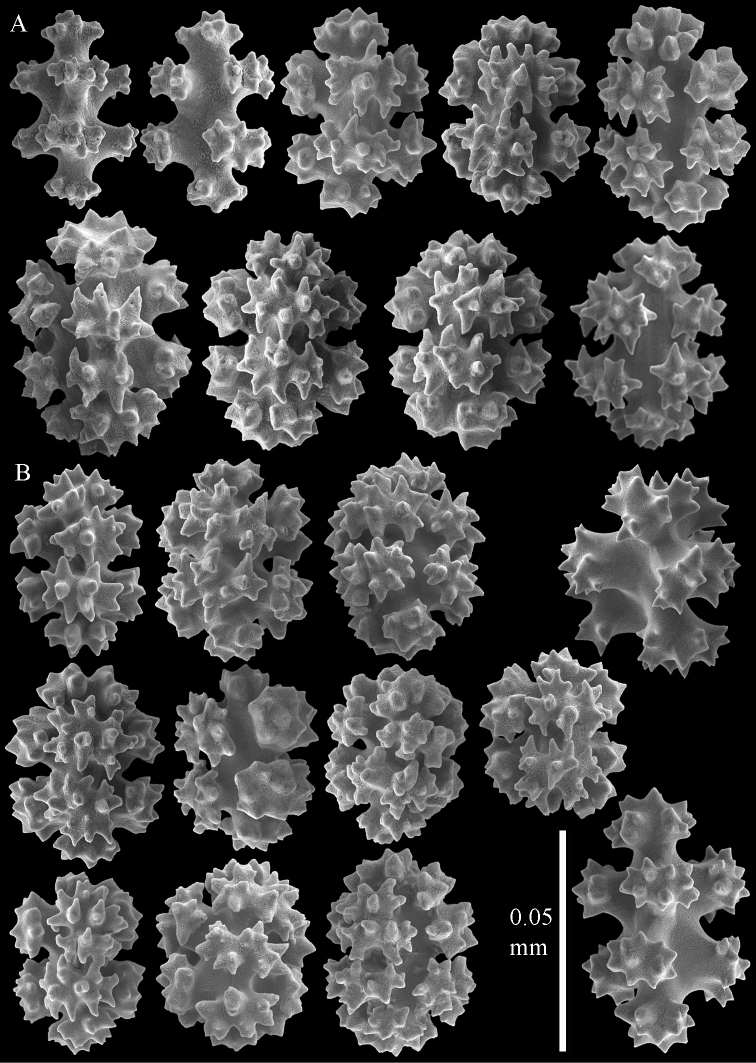
Sclerites of *Parasphaerasclera valdiviae* comb. n., RMNH Coel. 40206 **A** Surface layer of polyparium **B** Interior of polyparium.

**Figure 7.**
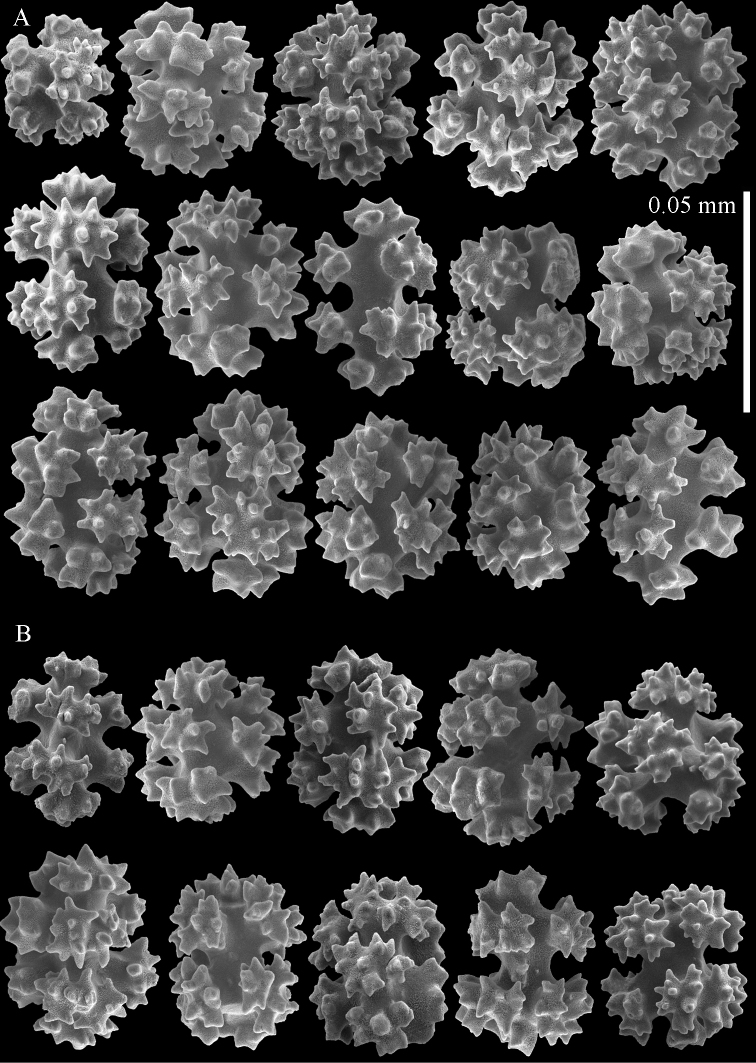
Sclerites of *Parasphaerasclera valdiviae* comb. n., RMNH Coel. 40206 **A** Surface layer of stalk **B** Interior of stalk.

**Figure 8.**
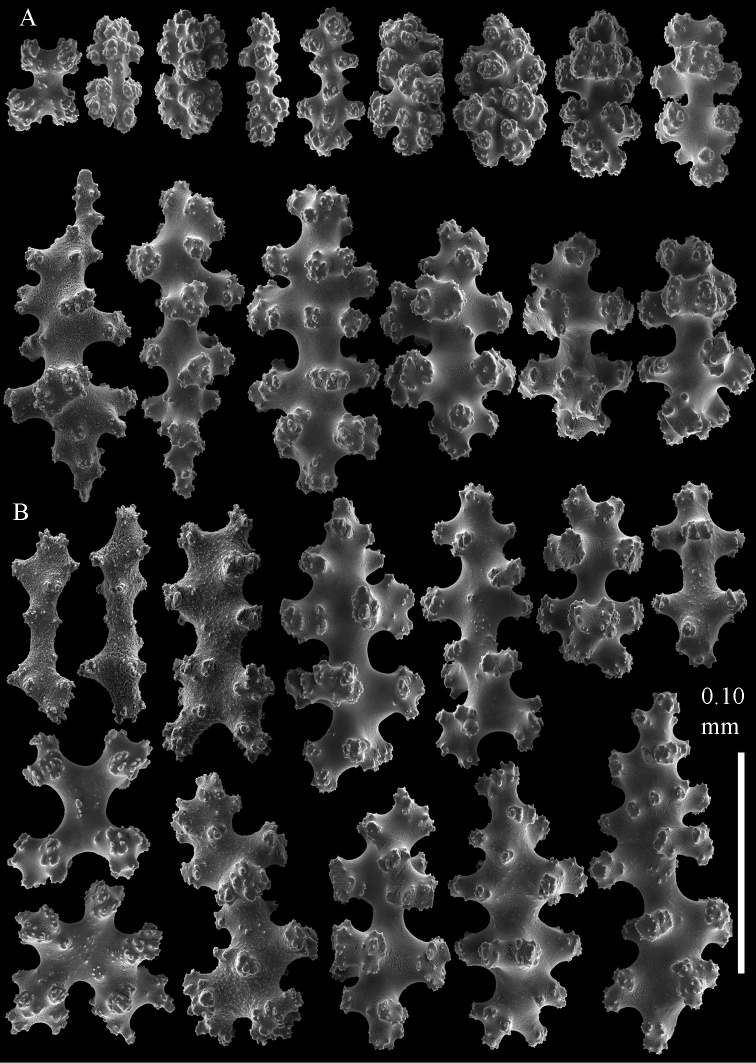
Sclerites of *Parasphaerasclera* aff. *grayi* comb. n. RMNH Coel. 40920 **A** Surface layer of polyparium **B** Interior of polyparium.

**Figure 9.**
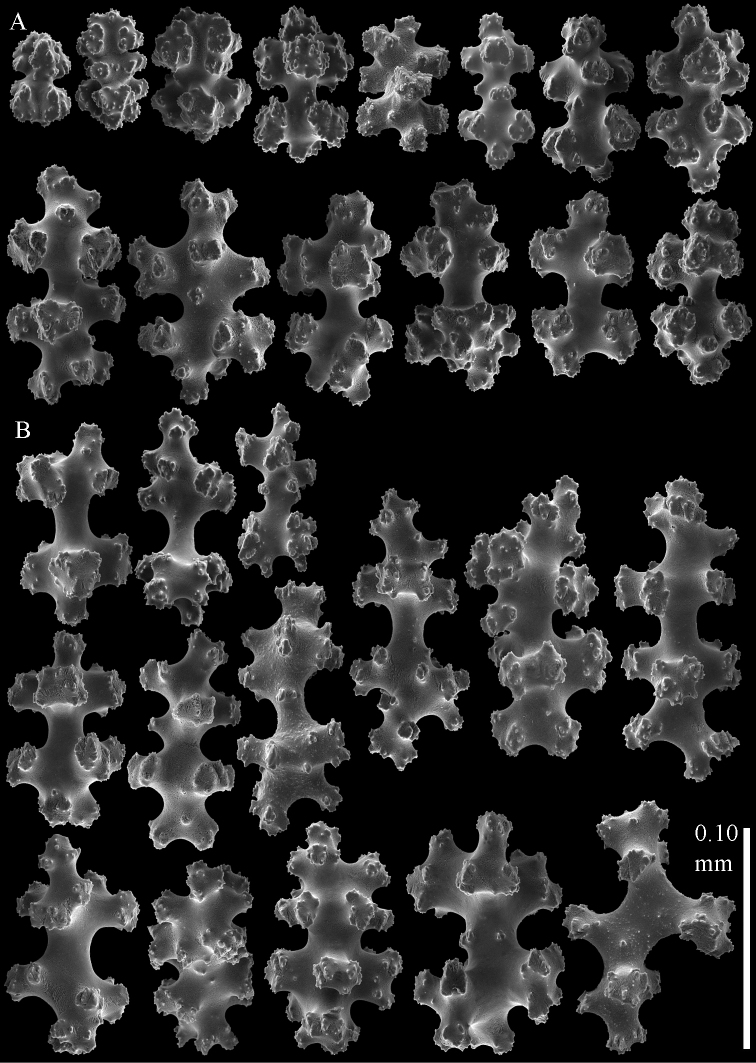
Sclerites of *Parasphaerasclera* aff. *grayi* comb. n. RMNH Coel. 40920 **A** Surface layer of stalk **B** Interior of stalk.

As diagnosed by Verseveldt and Bayer (1988) and modified by Williams (2003), the alcyoniid genus *Eleutherobia* Pütter, 1900 encompasses species with a diversity of sclerite forms and arrangements. Species within this genus are united primarily by their digitiform to lobular colony growth forms, although the diagnosis was recently emended to include capitate forms (Williams 2003). A subset of the species within *Eleutherobia* have in common a complete lack of sclerites in the polyps, and the sclerites in the surface and interior of the colony are predominantly small radiates and spheroids (Figs 4–9). Molecular analyses unite these species in a clade far removed phylogenetically from other genera of Alcyoniidae (Fig. 1), thus we describe a new family to accommodate them. Parasphaerascleridae fam. n. falls outside of the large Holaxonia–Alcyoniina clade of Octocorallia to which the majority of soft corals belong (McFadden et al. 2006). Although several other genera of Alcyoniidae also lie outside of Holaxonia–Alcyoniina (e.g., *Paraminabea*, *Anthomastus*, *Sphaerasclera* gen. n.), they are united with the scleraxonian families Coralliidae and Paragorgiidae in the *Anthomastus*-*Corallium* clade (McFadden et al. 2006). Paraphaerascleridae fam. n. does not belong to that clade.

#### Parasphaerasclera gen. n.

http://zoobank.org/F0625B3D-65FD-4B94-A535-2FFA46D84AD1

http://species-id.net/wiki/Parasphaerasclera

Figs 4 –9

##### Type species.

*Alcyonium rotiferum* Thomson, 1910, by original designation.

##### Diagnosis.

As for the family.

##### Etymology.

From the Greek root *para*-, meaning beside or alongside of *Sphaerasclera* gen. n. These two genera share similar sclerite complements and forms, and both were previously considered to belong to *Eleutherobia*. Gender: fem.

##### Remarks.

All species of *Eleutherobia* that have digitiform to digitate or lobate growth forms, lack polyp sclerites, and have radiates or spheroids in the colony surface and interior are hereby reassigned to *Parasphaerasclera* gen. n. These include *Parasphaerasclera albiflora* (Utinomi, 1957) comb. n., *Parasphaerasclera aurea* (Benayahu & Schleyer, 1995) comb. n., *Parasphaerasclera grayi* (Thomson & Dean, 1931) comb. n., *Parasphaerasclera nezdoliyi* (Dautova & Savinkin, 2009) comb. n., *Parasphaerasclera rotifera* (Thomson, 1910) comb. n., and *Parasphaerasclera zanahoria* (Williams, 2000) comb. n. Although molecular data to support their inclusion in this clade are only available for *Parasphaerasclera aurea*, *Parasphaerasclera* aff. *grayi* and *Parasphaerasclera rotifera*, the other three species all share the diagnostic morphological characters of the family (Utinomi 1957, Imahara 1977, Williams 2000b, Dautova and Savinkin 2009). These six species are morphologically distinct from the type species of *Eleutherobia*, *Eleutherobia rigida* (=*Eleutherobia japonica*, Pütter, 1900), which has polyp sclerites arranged as a collaret and points of spindles; radiates, spindles and club-like sclerites in the colony surface; and spindles in the interior coenenchyme (Verseveldt and Bayer 1988).

We also reassign two species of *Alcyonium* to *Parasphaerasclera* gen. n., *Parasphaerasclera morifera* (Tixier-Durivault, 1954) comb. n. and *Parasphaerasclera valdiviae* (Kükenthal, 1906) comb. n. The inclusion of *Parasphaerasclera valdiviae* comb. n. in *Parasphaerasclera* gen. n. is supported by both molecular phylogenetic (Fig. 1) and morphological evidence. *Parasphaerasclera valdiviae* comb. n. lacks polyp sclerites, the sclerites found in the surface of the colony are compact radiates and spheroids (Figs 6–7), and it shares with *Parasphaerasclera rotifera* comb. n. a growth form in which a conspicuous stalk gives rise to either branched or digitate lobes (Verseveldt and Williams 1988).

*Parasphaerasclera morifera* comb. n., another speciesfor which we lack molecular data,shares many morphological characters with other species of *Parasphaerasclera* gen. n. Verseveldt and Bayer (1988) synonymized *Nidalia morifera* Tixier-Durivault, 1954 with *Eleutherobia rotifera*, but Williams (1992) later maintained the distinction between them. He reassigned *Nidalia morifera* not to *Eleutherobia* but rather to *Alcyonium*, based on its lack of permanent calyces. Although Verseveldt and Bayer’s (1988) diagnosis of *Eleutherobia* stated “Anthocodiae retractile within calyces” (p. 27), Benayahu and Schleyer (1995) noted that some of the species included in the genus lacked permanent calyces. Williams and Little (2001) subsequently emended the diagnosis of *Eleutherobia* to “calyces absent, although retracted polyps often form low rounded to conspicuous protuberances” (p. 198). *Parasphaerasclera morifera* comb. n. is strikingly similar to *Parasphaerasclera aurea* comb. n.; both species have a digitiform to lobular growth form with a short, indistinct stalk, and the sclerites in the colony surface are compact radiates and spheroids (Figs 4A, 5A). The only difference between the two species is the presence of “double-deltoids” in the colony interior of *Parasphaerasclera aurea* comb. n. (Figs 4B, 5B) (Benayahu and Schleyer 1995).

It is possible that another South African species of *Alcyonium*, *Alcyonium distinctum* Williams, 1988, may also belong to this genus. Like other species of *Parasphaerasclera* gen. n. it lacks sclerites in the polyps, and the sclerites in the stalk surface are tuberculate spheroids and radiates (Williams 1988, 1992). The colony growth form is lobate, with lobes arising from a short, thick stalk, somewhat resembling *Parasphaerasclera valdiviae* comb. n. Unlike other species of *Parasphaerasclera* gen. n., however, in *Alcyonium distinctum* the sclerites are restricted to the stalk surface: there are no sclerites in the lobes (polypary) and interior of the colony (Williams 1988). In addition, the sclerites are not colored, and the bright purple color of living colonies is the result of an alcohol-soluble pigment. Material is not currently available for molecular analysis, so we cannot yet confirm the placement of *Alcyonium distinctum* in *Parasphaerasclera* gen. n.

A number of specimens of *Parasphaerasclera* gen. n. are known that differ somewhat from the descriptions of any of the nominal species listed above, and may represent additional, undescribed species of the genus. For example, there is considerable variation among those specimens of *Parasphaerasclera grayi* comb. n. that have been described and illustrated in the literature. Thomson and Dean’s original description (1931: 37) is rather confusing and sclerites were not depicted. The lectotype of *Parasphaerasclera grayi* that was designated and described by Verseveldt and Bayer (1988, figs. 24, 25) has sclerites in the colony surface that consist of 8-radiates (0.06–0.08 mm in length), crosses, and rods with tuberculate processes. The sclerites of the colony interior include particularly distinctive rod-like forms with smooth waists and high processes, up to 0.18 mm in length. Williams (2001) subsequently re-described *Parasphaerasclera grayi* based on specimens from the Solomon Islands. His specimens include 7- and 8-radiates (0.06–0.08 mm long) and crosses similar to those of the lectotype, but the rod-like sclerites in the colony interior are considerably smaller (most 0.07–0.08 mm long) and lack smooth waists (Williams 2001, figs 5–8). In contrast, specimens of *Parasphaerasclera grayi* from Vietnam that were later re-described and illustrated by Dautova and Savinkin (2009, figs. 5–7) include “rather smooth” (p. 10) rods that more closely resemble those depicted by Verseveldt and Bayer (1988), but are somewhat smaller, up to 0.14 mm long. The specimens from Palau that we have sequenced and identified here as *Parasphaerasclera* aff. *grayi* have tuberculate rods that lack a smooth waist (Figs 8–9), similar to those of Williams’s specimens. In our specimens, however, the rods are considerably larger (to 0.16 mm long). In addition, although the radiates in the colony surface of *Parasphaerasclera* aff. *grayi* from Palau are similar in size to those of other *Parasphaerasclera grayi* specimens, they are more compact and some approach ovals in form (Figs 8A, 9A). This range of variation in sclerite form and size observed among the different specimens attributed to *Parasphaerasclera grayi* suggests that more than one species may be involved. It remains to be determined if *Parasphaerasclera* aff. *grayi* from Palau is the same as *Parasphaerasclera grayi* *sensu* Williams (2001) from the Solomons, and if either of these forms is conspecific with Dautova and Savinkin’s (2009) material from Vietnam. The latter most closely matches the *Parasphaerasclera grayi* lectotype of Verseveldt and Bayer (1988).

*Parasphaerasclera* gen. n. is most similar morphologically to the alcyoniid genera *Paraminabea* and *Sphaerasclera* gen. n. All three genera lack sclerites in the polyps and have spheroids or radiates in the colony surface and interior. The polyps of *Paraminabea*, however, are dimorphic, while those of *Parasphaerasclera* gen. n. are monomorphic. The unique capitate growth form of *Sphaerasclera* gen. n. distinguishes it from all species of *Parasphaerasclera* gen. n., which are digitiform to digitate or lobate. *Parasphaerasclera* gen. n. is also easily distinguished from the alcyoniid genera *Eleutherobia* and *Alcyonium* *sensu stricto*, both of which have sclerites arranged to form a collaret and points in the polyps, and spindles or rods in the colony interior.

## Discussion

Following the taxonomic changes we have made here, eleven species remain in *Eleutherobia*. The morphological heterogeneity of these species and their similarities to some other genera suggest that further taxonomic revisions are likely to be necessary. Six of the remaining species of *Eleutherobia* have polyps with a distinct collaret and points of spindles; radiates, spindles and club-like sclerites in the colony surface; and spindles in the interior coenenchyme. These species likely belong to *Alcyonium* *sensu stricto.* Included among them is the type species of *Eleutherobia*, *Eleutherobia rigida* (=*Eleutherobia japonica*, Pütter, 1900), as well as *Eleutherobia grandiflora* (Kükenthal, 1906), *Eleutherobia rubra* (Brundin, 1896), *Eleutherobia somaliensis* Verseveldt & Bayer 1988, *Eleutherobia splendens* (Thomson & Dean, 1931), and *Eleutherobia unicolor* (Kükenthal, 1906). Another four species of *Eleutherobia* likewise have a collaret and points of spindles but have tuberculate radiates, spheroids and irregular forms in the colony surface, and spindle-like sclerites with narrow pointed ends and thick waists in the colony interior (Verseveldt and Bayer 1988). Included in this group are *Eleutherobia dofleini* (Kükenthal, 1906), *Eleutherobia duriuscula* (Thomson & Dean, 1931), *Eleutherobia flava* (Nutting, 1912), and possibly *Eleutherobia sumbawensis* Verseveldt & Bayer 1988. Whether these species also might belong to *Alcyonium* or to a different genus cannot be determined at present. *Eleutherobia vinadigitaria* Williams & Little, 2001, a species that has needle-like spindles in the polyps and no sclerites in the colony interior, is unlike any other species of *Eleutherobia*. Acquisition of material suitable for molecular phylogenetic analysis will greatly facilitate future efforts to determine the appropriate taxonomic placement of these species.

Until such time as the phylogenetic relationships among the species that remain in *Eleutherobia* can be determined, we modify the most recent diagnosis of the genus (Williams 2003) as follows:

Alcyoniid soft corals, usually digitiform (conical to cylindrical), sometimes digitate to lobate or clavate; polyparium arising from a common unbranched stalk. Polyps monomorphic. Calyces absent, although retracted polyps may form low rounded or mound-like protuberances of the coenenchyme. Anthocodial sclerites present, arranged in points or collaret and points. Coenenchymal sclerites mostly derived from radiates, although spindles, barrels, tuberculate spheroids, rod-like forms or crosses sometimes present. Color permanent and contained in the sclerites. Azooxanthellate.

## Conclusions

Molecular phylogenetic analyses of a number of species belonging to the alcyoniid genera *Eleutherobia* and *Alcyonium* have highlighted the heterogeneity of these two taxa as well as some overlap between them. As an initial effort to align the morphology-based taxonomic classification of species in these genera with molecular phylogenetic evidence of their evolutionary affinities we have reassigned two species of *Eleutherobia* with capitate growth forms, polyp sclerites arranged as a collaret and points, and spindles in the colony interior to *Alcyonium*; assigned a phylogenetically unique species with monomorphic polyps and a capitate growth form to a new genus of Alcyoniidae, *Sphaerasclera* gen. n.; and designated a new family, Parasphaerascleridae fam. n., and genus, *Parasphaerasclera* gen. n., to accommodate species with monomorphic polyps that lack sclerites in the polyps and have predominantly radiates and spheroids in the colony surface and interior. These are the first of many taxonomic revisions that will be required to reconcile the classification of genera currently assigned to Alcyoniidae with their phylogenetic relationships. Whereas the revisions we have made herein are well supported by both morphological and molecular phylogenetic evidence, considerably more evidence of both types will be necessary before we can begin to make taxonomic sense of the poorly resolved relationships among the many additional alcyoniid genera that belong to the Holaxonia–Alcyoniina clade of octocorals.

## Supplementary Material

XML Treatment for Sphaerasclera

XML Treatment for Parasphaerascleridae

XML Treatment for Parasphaerasclera

## Appendix

Taxa and sequences included in phylogenetic analysis. (doi: 10.3897/zookeys.346.6270.app) File format: Microsoft Excell file (xls). **Explanation note:** Taxa and sequences included in phylogenetic analysis. Museum accession numbers of sequenced specimens given when known. NA: no accession.

## References

[B1] Alderslade P (2000) Four new genera of soft corals (Coelenterata: Octocorallia), with notes on the classification of some established taxa. Zoologische Mededelingen Leiden 74: 237-249.

[B2] Bayer FM (1981) Key to the genera of Octocorallia exclusive of Pennatulacea (Coelenterata: Anthozoa), with diagnoses of new taxa. Proceedings of the Biological Society of Washington 94: 878-901.

[B3] Bayer FM (1993) Taxonomic status of the octocoral genus *Bathyalcyon* (Alcyoniidae: Anthomastinae), with descriptions of a new subspecies from the Gulf of Mexico and a new species of *Anthomastus* from Antarctic waters. Precious Corals & Octocoral Research 1: 3-13.

[B4] Benayahu Y Schleyer M (1995) Corals of the south-west Indian Ocean II. *Eleutherobiaaurea* spec. nov. (Cnidaria, Alcyonacea) from deep reefs on the KwaZulu-Natal coast, South Africa. Oceanographic Research Instititute Investigational Reports 68: 1-12.

[B5] Berntson EA Bayer FM McArthur AG France SC (2001) Phylogenetic relationships within the Octocorallia (Cnidaria: Anthozoa) based on nuclear 18S rRNA sequences. Marine Biology 138: 235-246. doi: 10.1007/s002270000457

[B6] Bouchet P Héros V Lozouet P Maestrati P (2008) A quarter-century of deep-sea malacological exploration in the South and West Paciﬁc: Where do we stand? How far to go? In: Héros V Cowie RH Bouchet P (Eds) Tropical Deep-Sea Benthos 25, vol 196: 9–40.

[B7] Brockman SA McFadden CS (2012) The mitochondrial genome of *Paraminabea aldersladei* (Cnidaria: Anthozoa: Octocorallia) supports intramolecular recombination as the primary mechanism of gene rearrangement in octocoral mitochondrial genomes. Genome Biology and Evolution 4: 882-894. doi: 10.1093/gbe/evs074 PMC3468961 22975720

[B8] Daly M Brugler MR Cartwright P Collins AG Dawson MN Fautin DG France SC McFadden CS Opresko DM Rodriguez E Romano S Stake J (2007) The phylum Cnidaria: A review of phylogenetic patterns and diversity 300 years after Linnaeus. Zootaxa 1668: 1-766.

[B9] Dautova TN Savinkin OV (2009) New data on soft corals (Cnidaria: Octocorallia: Alcyonacea) from Nha Trang Bay, South China Sea. Zootaxa 2027: 1-27.

[B10] Fabricius K Alderslade P (2001) Soft Corals and Sea Fans: a Comprehensive Guide to the Tropical Shallow-water Genera of the Central West-Pacific, the Indian Ocean and the Red Sea. Australian Institute of Marine Science, Townsville, 264 pp.

[B11] Imahara Y (1977) Alcyoniid octocorals from Suruga Bay, the Pacific coast of central Japan. Annotationes Zoologicae Japonenses 50: 31-35.

[B12] Katoh K Kuma K Toh H Miyata T (2005) MAFFT version 5: improvement in accuracy of multiple sequence alignment. Nucleic Acids Research 33: 511-513. doi: 10.1093/nar/gki198 15661851 PMC548345

[B13] McFadden CS Ofwegen LP van (2012) Stoloniferous octocorals (Anthozoa, Octocorallia) from South Africa, with descriptions of a new family of Alcyonacea, a new genus of Clavulariidae, and a new species of *Cornularia* (Cornulariidae). Invertebrate Systematics 26: 331–356. doi: 10.1071/IS12035

[B14] McFadden CS Ofwegen LP van (2013) A second, cryptic species of the soft coral genus *Incrustatus* (Anthozoa: Octocorallia: Clavulariidae) from Tierra del Fuego, Argentina, revealed by DNA barcoding. Helgoland Marine Research 67: 137-147. doi: 10.1007/s10152-012-0310-7

[B15] McFadden CS Donahue RM Hadland BK Weston R (2001) A molecular phylogenetic analysis of reproductive trait evolution in the soft coral genus *Alcyonium*. Evolution 55: 29-42. doi: 10.1093/icb/icq056 11263746

[B16] McFadden CS France SC Sánchez JA Alderslade P (2006) A molecular phylogenetic analysis of the Octocorallia (Coelenterata: Anthozoa) based on mitochondrial protein-coding sequences. Molecular Phylogenetics and Evolution 41: 513-527. doi: 10.1016/j.ympev.2006.06.010 16876445

[B17] McFadden CS Sánchez JA France SC (2010) Molecular phylogenetic insights into the evolution of Octocorallia: a review. Integrative and Comparative Biology 50: 389-410. 21558211 10.1093/icb/icq056

[B18] McFadden CS Benayahu Y Pante E Thoma JN Nevarez PA France SC (2011) Limitations of mitochondrial gene barcoding in the cnidarian sub-class Octocorallia. Molecular Ecology Resources 11: 19-31. doi: 10.1111/j.1755-0998.2010.02875.x 21429097

[B19] Ofwegen LP van Häussermann V Försterra G (2007) The genus *Alcyonium* (Octocorallia: Alcyonacea: Alcyoniidae) in Chile. Zootaxa 1607: 1-19.

[B20] Posada D Crandall KA (1998) Modeltest: testing the model of DNA substitution. Bioinformatics 14: 817–818. doi: 10.1093/bioinformatics/14.9.817 9918953

[B21] Ronquist F Teslenko M van der Mark P Ayres D Darling A Höhna S Larget B Liu L Suchard MA Huelsenbeck JP (2012) MrBayes 3.2: Efficient Bayesian phylogenetic inference and model choice across a large model space. Systematic Biology 61: 539-542. doi: 10.1093/sysbio/sys029 22357727 PMC3329765

[B22] Thomson JA Dean LMI (1931) The Alcyonacea of the Siboga Expedition with an addendum to the Gorgonacea. Siboga Expedition Monograph 13d: 1–227.

[B23] Thomson JS (1910) The Alcyonaria of the Cape of Good Hope and Natal. Alcyonacea. Transactions of the Royal Society of Edinburgh XLVII, part III: 549–589, pls I–IV.

[B24] Utinomi H (1957) The alcyonarian genus *Bellonella* from Japan, with descriptions of two new species. Publications of the Seto Marine Biological Laboratory 6: 147–168, pls. 9–10.

[B25] Verseveldt J Bayer FM (1988) Revision of the genera *Bellonella*, *Eleutherobia*, *Nidalia* and *Nidaliopsis* (Octocorallia: Alcyoniidae and Nidaliidae), with descriptions of two new genera. Zoologische Verhandelingen Leiden 245: 1-131.

[B26] Verseveldt J Williams GC (1988) A redescription of the soft coral *Alcyonium valdiviae* Kükenthal, 1906, with the description of a new species of *Litophyton* Førskal, 1775, from southern Africa (Octocorallia, Alcyonacea). Annals of the South African Museum 97: 315–328.

[B27] Williams GC (1986) Morphology, systematics, and variability of the southern African soft coral *Alcyonium variabile* (J. Stuart Thomson, 1921) (Octocorallia, Alcyoniidae). Annals of the South African Museum 96: 241-270.

[B28] Williams GC (1988) Four new species of southern African octocorals (Cnidaria: Alcyonacea), with a further diagnostic revision of the genus *Alcyonium* Linnaeus, 1758. Zoological Journal of the Linnean Society 92: 1-26. doi: 10.1111/j.1096-3642.1988.tb01524.x

[B29] Williams GC (1992) The Alcyonacea of Southern Africa. Stoloniferous octocorals and soft corals (Coelenterata, Anthozoa). Annals of the South African Museum 100: 249-358.

[B30] Williams GC (2000a) Two new genera of soft corals (Anthozoa: Alcyoniidae) from South Africa, with a discussion of diversity and endemism in the southern African octocorallian fauna. Proceedings of the California Academy of Sciences 52: 65-75.

[B31] Williams GC (2000b) A new species of the soft coral genus *Eleutherobia* Pütter, 1900 (Coelenterata: Alcyoniidae) from the Tonga Islands. Proceedings of the California Academy of Sciences 52: 159-169.

[B32] Williams GC (2001) First record of a bioluminescent soft coral: description of a disjunct population of *Eleutherobia grayi* (Thomson and Dean, 1921) from the Solomon Islands, with a review of bioluminescence in the Octocorallia. Proceedings of the California Academy of Sciences 52: 209-225.

[B33] Williams GC (2003) Capitate taxa of the soft coral genus *Eleutherobia* (Octocorallia: Alcyoniidae) from Palau and South Africa; a new species and a new combination. Zoologische Verhandelingen Leiden 345: 419-436.

[B34] Williams GC Alderslade P (1999) Revisionary systematics of the western Pacific soft coral genus *Minabea* (Octocorallia: Alcyoniidae), with descriptions of a related new genus and species from the Indo-Pacific. Proceedings of the California Academy of Sciences 51: 337-364.

[B35] Williams GC Cairns S (2013) Systematic list of valid octocoral genera. http://research.calacademy.org/redirect?url=http://researcharchive.calacademy.org/research/izg/orc_home.html [accessed 29 August 2013]

[B36] Williams GC Little SA (2001) A new species of the soft coral genus *Eleutherobia* Pütter, 1900 (Octocorallia: Alcyoniidae) from South Africa. Proceedings of the California Academy of Sciences 52: 195-208.

[B37] Zwickl DJ (2006) Genetic algorithm approaches for the phylogenetic analysis of large biological sequence datasets under the maximum likelihood criterion. Ph.D. thesis, University of Texas, Austin, Texas.

